# Effect of atropine on time to cardiac arrest in hypoxic bradycardia: a randomised cross-over study in a porcine apnoea model

**DOI:** 10.1016/j.bjao.2026.100565

**Published:** 2026-06-11

**Authors:** Tomáš Pařízek, Petr Waldauf, Michal Kalina, Zuzana Kurzová, Irena Odstrčilová, Jan Hunák, Jakub Freml, Radim Dvořák, David Peřan, Alena Vlková, František Duška

**Affiliations:** 1 PIGILAB, Department of Anaesthesia and Intensive Care Medicine, Third Faculty of Medicine, Charles University, Prague and University Hospital Kralovske Vinohrady, Prague, Czech Republic; 2 Faculty of Medicine in Hradec Kralove, Charles University, Hradec Kralove, Czech Republic; 3 Centre of Toxicology and Health Safety, National Institute of Public Health, Prague, Czech Republic

**Keywords:** apnoea, atropine, bradycardia, cardiac arrest, hypoxia, swine

## Abstract

**Background:**

In profound hypoxia, including ‘cannot intubate, cannot oxygenate’ (CICO) situations, bradycardia may represent an oxygen-conserving reflex rather than a correctable pathology. We hypothesised that atropine would, by increasing heart rate and myocardial oxygen demand, hasten cardiac arrest in apnoea-induced hypoxic bradycardia.

**Methods:**

We performed a randomised, placebo-controlled, cross-over trial in intubated, anaesthetised pigs (*n*=8). Each animal underwent two episodes of apnoea-induced hypoxia separated by a 6–12-h stabilisation period. At the onset of hypoxia-induced bradycardia, 1 mg of atropine or placebo was administered in random order. Apnoea was maintained until 50 s after cardiac arrest, then mechanical ventilation was resumed, and standard cardiopulmonary resuscitation was initiated. The primary outcome was time from study-drug administration to cardiac arrest. Secondary outcomes included time to return of spontaneous circulation (ROSC), heart-rate response, oxygen-delivery indices, and brain tissue oxygen tension.

**Results:**

No statistically significant difference in time to cardiac arrest was detected: 142 seconds (95% Confidence interval [CI] 84–199) after atropine *vs* 138 seconds (95% CI 80–195) after placebo; mean difference 4 seconds (95% CI −33 to 41), *P*=0.803. In four animals, atropine shortened the time to arrest, and in four it prolonged it. No significant between-condition differences were observed in systemic haemodynamic, brain oxygenation, lactate concentrations, or time to ROSC.

**Conclusions:**

In this porcine model of apnoea-induced hypoxic bradycardia, atropine neither delayed cardiac arrest nor improved oxygenation, perfusion, or resuscitation outcomes.

**Trial registration number:**

Open Science Framework (OSF), DOI: https://doi.org/10.17605/OSF.IO/492NY.

**Editor’s key points**

• In a porcine model of apnoea-induced hypoxic bradycardia, atropine provided no benefit over placebo.
• Atropine tended to increase heart rate but did not improve oxygen delivery or prolong time to cardiac arrest.
• Findings do not support routine atropine use for hypoxic bradycardia in comparable settings (e.g. cannot intubate, cannot oxygenate).
• Bradycardia is a manifestation of hypoxia in this context; treating hypoxia is the priority.

Bradycardia is a common, time-critical sign during severe hypoxia and is frequently encountered in prearrest states, including ‘cannot intubate, cannot oxygenate’ (CICO) scenarios. Current ACC/AHA/HRS guidelines and European Resuscitation Council guidelines 2025 recommend intravenous atropine as the first-line pharmacotherapy for symptomatic bradycardia in adults, while noting that the benefit is uncertain when bradycardia is due to hypoxia or ischaemia; paediatric guidance advises against atropine unless bradycardia is vagally mediated.^1^, ^2^, ^3^

Atropine increases heart rate by antagonising muscarinic acetylcholine receptors; the clinical rationale is that raising heart rate (and thus cardiac output) might augment oxygen delivery. It is also unclear whether increased vagal tone may be a contributing factor to cardiac arrest during hypoxia. On the other hand, the slowing of heart rate may be part of an evolutionarily conserved oxygen-conserving reflex mediated via carotid body chemoreceptors and enhanced vagal output.^4^^,^^5^ This reflex bradycardia reduces myocardial oxygen consumption and prolongs the diastole, during which the myocardium is perfused. In turn, bradycardia may protect the heart during transient oxygen deprivation. Two rodent studies suggest that, in the setting of sustained hypoxia, preventing this reflex (e.g. via vagotomy or atropine) can lead to a paradoxical acceleration toward cardiac arrest due to increased myocardial oxygen demand.^6^^,^^7^

The trade-off between the potential gains in cardiac output and the risk of hastening myocardial exhaustion remains largely theoretical and untested in large animals. To address this gap, we conducted a randomised, placebo-controlled, crossover study in instrumented juvenile pigs to test whether intravenous atropine alters time to cardiac arrest (primary outcome) during apnoea-induced hypoxic bradycardia.

## Methods

### Trial design

This was a single-centre, randomised, placebo-controlled, crossover study in female domestic pigs. Each animal served as its own control, receiving both interventions (intravenous atropine and placebo) in a randomised sequence, separated by a predefined stabilisation/washout period to allow full haemodynamic recovery (6–12 h). The allocation ratio for the first period was 1:1. No changes to the trial methods were made after study commencement.

### Participants

Healthy, farm-bred, female pigs (30–50 kg) were eligible. Animals would have been excluded had they exhibited pre-existing cardiovascular abnormalities, persistent arrhythmias, or any adverse events during instrumentation that would preclude protocol completion.

### Setting and oversight

Experiments were performed in the certified preclinical research laboratory of the National Institute of Public Health (ID: MZE-62185/2022-13143; Prague, Czech Republic), equipped for large-animal anaesthesia, monitoring, and intensive post-resuscitation care. The protocol was approved by the Ministry of Health (ID: MZDR 23680/2024-5/OVZ, serial number 48/2024) and conducted in accordance with ARRIVE guidelines.^8^ All procedures, including instrumentation and euthanasia, were performed under deep anaesthesia by personnel certified in animal care and welfare. The study was prospectively registered on the Open Science Framework (OSF, registration: https://osf.io/492ny/).

### Interventions

Animals were premedicated with intramuscular ketamine 20 mg kg^-1^ and azaperone 2 mg kg^-1^, followed by intravenous propofol 1–2 mg kg^-1^ bolus and fentanyl 5 μg kg^-1^. After endotracheal intubation, they were mechanically ventilated in volume-controlled mode (FiO_2_ 0.4, with minute ventilation titrated to maintain EtCO_2_ = 4.8 kPa). Anaesthesia was maintained with continuous infusion of propofol 4–8 mg kg^-1^ h^-1^ and fentanyl 5–20 μg kg^-1^ h^-1^; neuromuscular blockade was provided by rocuronium 2.5–5 mg kg^-1^ h^-1^. Invasive monitoring included femoral venous and arterial catheters, a balloon-tipped pulmonary artery (Swan–Ganz) catheter via the internal jugular vein, and an intraparenchymal PbtO_2_ probe (Neurovent-PTO 2L, Raumedic, Germany) inserted through a frontal burr hole. Baseline measurements were obtained after normalisation of vital signs and arterial blood gases (including lactate). To attenuate sympathetic effects and standardise autonomic tone, all animals received metoprolol 1 mg kg^-1^ i.v. at baseline.^9^^,^^10^

**Fig 1:**
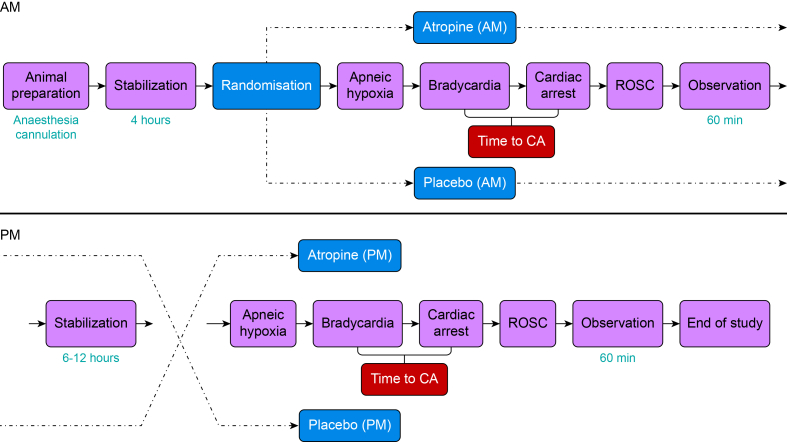
Study schematic. The AM panel shows Period 1: after anaesthesia and a 4-h stabilisation, animals were randomised 1:1 to atropine 1 mg i.v. or placebo i.v. at the onset of apnoea-induced bradycardia (T_0_). In accordance with the guideline-recommended dose of 0.5 mg of atropine for the treatment of bradycardia, and following the allometric dose conversion principles outlined by Nair and Jacob,^11^ we elected to administer 1 mg of atropine. The primary outcome, time to cardiac arrest (CA), was measured from T_0_. Following CA, CPR was initiated to ROSC and animals were observed for 60 min. The PM panel shows Period 2 after a 6–12 h stabilisation/washout with full haemodynamic recovery, when animals crossed over to the alternate treatment and the same procedure was repeated. CPR, cardiopulmonary resuscitation; ROSC, return of spontaneous circulation.

Hypoxia was induced by cessation of mechanical ventilation (apnoea). Upon development of bradycardia (heart rate <80 bpm, or >25% decrease from baseline if accompanied by hypotension), a single intravenous bolus of either atropine 1 mg or isotonic saline (placebo) was administered according to the randomised sequence. Animals were continuously monitored until cardiac arrest, predefined as asystole, ventricular fibrillation or an organised rhythm with a mean arterial pressure <40 mmHg for ≥30 s. Fifty seconds after arrest onset, cardiopulmonary resuscitation (CPR) was initiated according to a standardised protocol, aligned with ERC guidance (ventilation with FIO_2_ 1.0 and chest compressions 100–120 min^-1^).^2^ After return of spontaneous circulation (ROSC), animals remained anaesthetised, ventilated, normothermic and received fluids/vasopressors as required. Following full recovery (unsupported haemodynamics and arterial lactate < 2 mmol L^-1^), but no earlier than 6 h after the first cardiac arrest, animals crossed over to the alternate treatment under identical procedures (Fig. 1).^11^

### Outcomes

The primary outcome was time from study-drug administration (T_0_) to cardiac arrest, where T_0_ denotes the intravenous bolus of atropine 1 mg or placebo given at the onset of apnoea-induced bradycardia. Cardiac arrest was predefined as asystole, ventricular fibrillation, or an organised rhythm with mean arterial pressure <40 mmHg for ≥30 seconds.

Secondary outcomes were: (i) time from apnoea onset to cardiac arrest; (ii) heart-rate response after the first dose (change from predose baseline to the postdose peak within the first 60 seconds); and (iii) time to ROSC, defined as sustained MAP ≥60 mmHg with an organised rhythm for ≥60 seconds without chest compressions.

Exploratory outcomes included: (i) pulmonary artery occlusion pressure as a marker of diastolic function; (ii) cardiac output and oxygen delivery (from thermodilution and arterial/venous gases); and (iii) brain tissue oxygen tension (PbtO_2_).

No changes were made to the predefined primary or secondary outcomes after the trial initiation. All variables were captured continuously or at protocol-defined intervals and exported from the digital monitoring system for analysis.

### Sample size

Assuming a paired (crossover) comparison of the within-animal difference in time from study-drug administration to cardiac arrest (Atropine − Placebo), with an expected mean difference of 20% and a standard deviation (SD) of the paired differences of 15%, a two-sided α=0.05 test provides >80% power with *n*=6 animals (pairs). To allow for procedural losses, we planned to enrol up to 10 pigs; eight completed both periods and were included in the per-protocol analysis. In a balanced 2×2 cross-over design, the LMEM treatment contrast with a random intercept for subject is algebraically equivalent to the mean of paired within-subject differences; sample size can therefore be calculated from the variance of those differences, as was done here.

No interim analyses or stopping rules were specified, as each animal received both treatments and served as its own control.

### Randomisation

Randomisation was performed using a computer-generated random sequence in blocks of two to ensure a 1:1 balance of the first intervention (atropine *vs* placebo) across animals. The alternate treatment was administered in Period 2 after stabilisation/washout.

### Blinding

Investigators were not blinded to allocation; study drugs were administered open-label. Blinding was not feasible due to the small study team size.

### Statistical methods

Data from the 2×2 crossover experiment were analysed using linear mixed-effects models (LMEMs) in R (v4.5.1; R Core Team, Vienna, Austria) with the packages lme4, lmerTest, and emmeans. Each pig served as its own control via a random intercept. Model-selection algorithm (treatment-effect analyses). For each endpoint, we started from the most complex prespecified model and iteratively removed terms, comparing nested models by likelihood-ratio tests under maximum likelihood (ML). We retained the simplest model that was not worse than a more complex alternative. In our data, this procedure consistently selected the simplest model.

#### Primary outcome

For ‘time from study-drug administration (T_0_) to cardiac arrest’, the initial fixed-effects structure included treatment (atropine *vs* placebo), period (AM *vs* PM), and their interaction, with a random intercept for pig. Following the step-down procedure, the final model retained treatment as the only fixed effect (random intercept for pig); no period or carry-over term was included.

#### Secondary outcomes

The same step-down procedure was applied to each outcome. Unless specified otherwise, the selected model was reduced to treatment as the sole fixed effect with a random intercept for pig.

#### Descriptive period contrasts (AM vs PM)

For the baseline/physiology table, period effects were summarised from an LMEM with period as the only fixed effect and a random intercept for pig. We report mean (sd) by period, marginal means with 95% CI, and the mean difference (AM − PM) with 95% CI and the LMEM *P*-value.

#### Estimation and reporting

Variance components were estimated by restricted maximum likelihood REML (ML used only for model comparisons). Marginal means and contrasts were obtained with emmeans using equal weighting across cells. Effects are reported as mean differences with 95% CI; *P-*values are two-sided with α=0.05.

#### Descriptive statistics

Continuous variables are reported as mean (sd) unless noted otherwise; categorical variables as counts (percentages).

No subgroup or adjusted analyses were planned, as each animal acted as its own control in the cross-over design.

With *n*=8 pairs, this study was powered as an exploratory experiment and not for equivalence testing; the wide confidence intervals should be interpreted accordingly.

## Results

**Fig 2:**
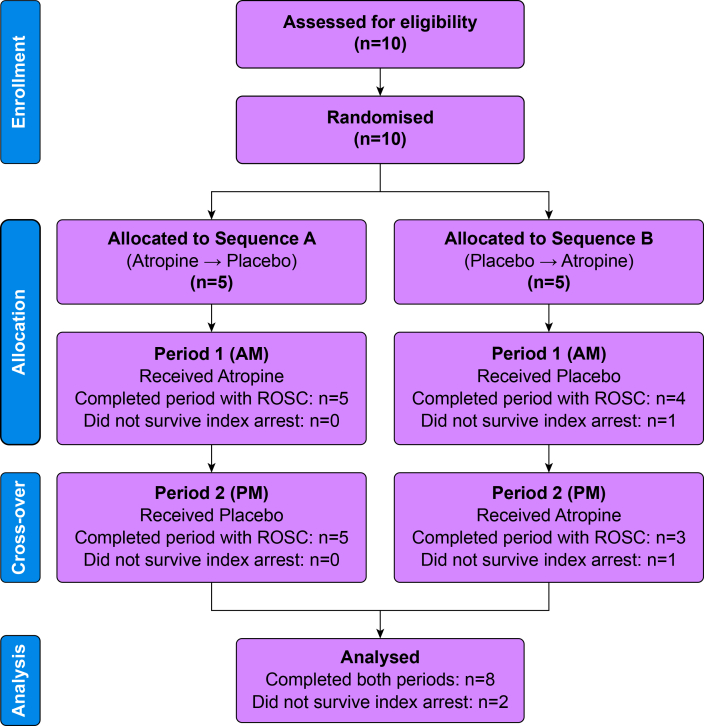
Study flow. Ten pigs were randomised 1:1 to Sequence A (Atropine → Placebo, *n*=5) or Sequence B (Placebo → Atropine, *n*=5). Eight completed both periods and entered the per-protocol analysis. Two died following the first arrest (placebo *n*=1 in Period 1; atropine *n*=1 in Period 2). ROSC, return of spontaneous circulation.

Of the 10 enrolled pigs, eight completed both periods and were included in the per-protocol analysis. Two animals died following the first cardiac arrest—one during the placebo period and one during the atropine period—and were excluded from the per-protocol set. Participant flow is shown in Fig. 2.

Baseline characteristics of the per-protocol population immediately before induction are shown in Table 1. Compared with AM, PM sessions showed modest but statistically significant reductions in cardiac output and arterial lactate, slightly lower body temperature and pH, and higher *P*CO_2_. In addition, central venous pressure (CVP) and intracranial pressure (ICP) were higher during PM, and brain tissue oxygen tension (PbtO_2_) was also higher in PM. Other variables (HR, MAP, SPAP/DPAP, PCWP, SaO_2_, EtCO_2_, DO_2_, pO_2_, haemoglobin) did not differ meaningfully between periods.

**Table 1:** Baseline physiological variables by period (AM *vs* PM). Values are mean (SD). Mean differences (AM − PM) with 95% CI and *P*-values were estimated using a linear mixed-effects model with a fixed effect for period only and a random intercept for animal (Pig ID); *N*=8 per period with the same animals measured in both periods. Weight is descriptive only. 95% CI is reported as ‘lower; upper’. BT, body temperature; CI, confidence interval; CO, cardiac output; CVP, central venous pressure; DO_2_, oxygen delivery; DPAP, diastolic pulmonary artery pressure; EtCO_2_, end-tidal carbon dioxide; Hb, haemoglobin; ICP, intracranial pressure; MAP, mean (systemic) arterial pressure; PCWP, pulmonary capillary wedge pressure; PbtO_2_, brain tissue oxygen tension; *P*CO_2_*,* arterial carbon dioxide partial pressure; pO_2_, arterial oxygen partial pressure; SaO_2_, arterial oxygen saturation; SPAP, systolic pulmonary artery pressure; SD, standard deviation. All pressures in mmHg.

Parameter	Period 1 (AM) mean (SD) (*N*=8)	Period 2 (PM) mean (SD) (*N*=8)	Mean difference (AM - PM) with 95% CI	*P*-value
Weight (kg)	37.4 (3.0)			N/A
HR (min^−1^)	81 (8.4)	75.4 (5.3)	6.0 (−0.4 to 12.4)	0.0627
MAP (mmHg)	72.1 (9.5)	80.2 (11.2)	−7.3 (−19.1 to 4.5)	0.1817
SPAP (mmHg)	30.8 (3.8)	30.8 (4.1)	0.1 (−5.1 to 5.4)	0.9522
DPAP (mmHg)	15.1 (2.2)	17.5 (1.6)	−2.4 (−4.9 to 0.1)	0.0597
CVP (mmHg)	10.8 (2.1)	11.9 (2.4)	−1.2 (−2.4 to −0.1)	0.0361
PCWP (mmHg)	11.8 (2.3)	12.9 (2.7)	−1.3 (−3.5 to 0.9)	0.1920
SaO_2_ (%)	99.7 (0.6)	99.8 (0.4)	−0.2 (−0.8 to 0.5)	0.5797
EtCO_2_ (kPa)	4.83 (0.12)	4.81 (0.31)	−0.02 (−0.19 to 0.16)	0.8574
Body temperature (°C)	38.9 (0.6)	38.7 (0.5)	0.3 (0.1–0.5)	0.0226
CO (L min ^−1^)	4.1 (0.9)	3.5 (0.5)	0.7 (0.0–1.3)	0.0416
DO_2_ (ml min ^−1^)	466.4 (159.7)	379.1 (69)	80.4 (−36.1 to 196.9)	0.1423
pH	7.53 (0.03)	7.49 (0.02)	0.04 (0.01–0.07)	0.0132
*P*O_2_ (kPa)	19.6 (3)	17.5 (2.5)	1.9 (−1.0 to 4.7)	0.1598
*P*CO_2_ (kPa)	5 (0.4)	5.4 (0.2)	−0.5 (−0.8 to −0.1)	0.0170
Lactate (mmol L^−1^)	1.3 (0.4)	0.8 (0.3)	0.6 (0.2–0.9)	0.0049
Hb (g l ^−1^)	82.6 (12.4)	80.8 (7.5)	0.7 (−9.3 to 10.7)	0.8694
ICP (mmHg)	17.9 (2)	22.1 (3.7)	−4.5 (−6.4 to −2.5)	0.0014
*P*btO_*2*_ (mmHg)	14.2 (15.4)	31.3 (11.6)	−17.5 (−23.5 to −11.5)	0.0004

Mean time from study-drug administration to cardiac arrest was 139.5 s (95% CI: 81.6–197.4) in the AM period *vs* 139.5 s (95% CI: 81.6–197.4) in the PM period; the mean difference (AM − PM) was 0.0 s (95% CI: −42.2 to 42.2); *P*=1.0.

### Immediate response to intervention

Table 2 summarises physiological changes from immediately before drug administration to 30 seconds afterwards. Heart rate increased numerically after atropine (mean difference 30 seconds − prior 44.0 [95% CI: −14.8 to 102.8], *P*=0.112) and after placebo (17.0 [−5.1 to 39.1], *P*=0.100), with no between-group difference at 30 seconds (28.0 [−35.7 to 91.7], *P*=0.315). Systemic (MAP) and pulmonary artery (SPAP, DPAP) pressures showed no consistent within-group changes; the only between-group difference was in DPAP at 30 s (−5.9 [−9.7; −2.2], p = 0.015). pH fell slightly in both arms (atropine −0.03 [−0.05 to −0.01], *P*=0.010; placebo −0.03 [−0.05 to −0.01], *P*=0.0065). *P*CO_2_ rose with placebo (0.5 kPa [0.2–0.8], *P*=0.0042) but not with atropine (0.1 [−0.5 to 0.6], *P*=0.788). Lactate increased modestly after atropine (0.5 mmol L^-1^ [0.0–1.0], *P*=0.042), while PbtO_2_ and ICP did not change significantly within or between groups. Overall, aside from the placebo-associated rise in *P*CO_2_ and a small lactate increase after atropine, short-term (30 seconds) responses were similar between atropine and placebo.

**Table 2:** Physiological parameters immediately before and 30 s after administration of atropine or placebo. Data are mean (SD). Within-treatment change (30 s − prior) and between-group difference at 30 s were estimated by LMEMs (random intercept for pig) and reported as mean difference with 95% CI and *P* (95% CI shown as ‘lower; upper’). Variables missing *vs*Table 1 were not analysed due to missing data. 95% CI is reported as ‘lower; upper’. BT, body temperature; CI, confidence interval; CO, cardiac output; CVP, central venous pressure; DO_2_, oxygen delivery; EtCO_2_, end-tidal carbon dioxide; Hb, haemoglobin; ICP, intracranial pressure; LMEM, linear mixed-effects model; PbtO_2_, brain tissue oxygen tension; PCWP, pulmonary capillary wedge pressure; *P*CO_2_, arterial carbon dioxide partial pressure; pO_2_, arterial oxygen partial pressure; SaO_2_, arterial oxygen saturation.

Parameter	Atropine				Placebo				Between groups at 30 s (Atropine − Placebo)	
	Immediately prior to administration mean (SD)	Thirty seconds after administration mean (SD)	LMEM mean difference (30 s − prior) with 95% CI	*P*	Immediately prior to administration mean (SD)	Thirty seconds after administration mean (SD)	LMEM mean difference (30 s − prior) with 95% CI	*P*	LMEM mean difference with 95% CI	*P*
HR (min^-1^)	63.1 (13.2)	105.0 (49.6)	44.0 (−14.8 to 102.8)	0.1123	65.8 (18.4)	77.0 (24.7)	17.0 (−5.1 to 39.1)	0.1000	28.0 (−35.7 to 91.7)	0.3152
SPAP (mmHg)	40.3 (15.6)	31.6 (9.5)	−7.2 (−24.6 to 10.2)	0.3385	36.9 (10.4)	38.0 (14.4)	4.7 (−0.0 to 9.5)	0.0500	−10.5 (−23.7 to 2.7)	0.0886
MAP (mmHg)	57.5 (28.8)	56.5 (42.3)	−4.5 (−17.8 to 8.8)	0.4238	44.9 (22.1)	56.6 (34.3)	5.6 (−22.5 to 33.7)	0.6092	11.4 (−11.3 to 34.0)	0.2135
DPAP (mmHg)	26.3 (8.2)	19.4 (5.9)	−4.8 (−10.6 to 0.9)	0.0821	23.2 (6.4)	24.8 (7.5)	3.5 (−0.3 to 7.3)	0.0605	−5.9 (−9.7 to −2.2)	0.0148
CVP (mmHg)	22.6 (4.3)	23.0 (2.3)	−1.2 (−4.2 to 1.9)	0.3737	20.8 (4.2)	21.5 (4.2)	0.7 (−1.0 to 2.4)	0.3632	1.5 (−3.7 to 6.7)	0.5022
PCWP (mmHg)	19.8 (4.6)	23.0 (8.4)	−3.0 (−47.9 to 41.9)	0.5520	19.4 (10.1)	19.0 (3.4)	2.0 (−61.5 to 65.5)	0.7578	3.8 (−9.3 to 16.8)	0.4758
SaO_2_ (%)	8.6 (5.9)	10.2 (6.6)	0.9 (−5.3 to 7.1)	0.7385	12.8 (8.8)	9.7 (6.3)	−4.5 (−10.7 to 1.8)	0.1336	0.5 (−7.9 to 9.0)	0.8819
pH	7.30 (0.06)	7.28 (0.04)	−0.03 (−0.05 to −0.01)	0.0104	7.30 (0.06)	7.27 (0.05)	−0.03 (−0.05 to −0.01)	0.0065	0.01 (−0.03 to 0.05)	0.6305
*P*O_2_ (kPa)	2.3 (0.7)	2.5 (0.5)	0.2 (−0.8 to 1.2)	0.6795	2.6 (0.8)	2.4 (0.6)	−0.3 (−1.1 to 0.6)	0.4226	−0.0 (−0.8 to 0.8)	0.9541
*P*CO_2_ (kPa)	8.7 (1.1)	8.8 (1.0)	0.1 (−0.5 to 0.6)	0.7838	8.6 (1.0)	9.0 (0.8)	0.5 (0.2–0.8)	0.0042	−0.3 (−1.4 to 0.9)	0.5717
Lactate (mmol L^-1^)	3.1 (1.5)	3.6 (1.2)	0.5 (0.0–1.0)	0.0417	3.1 (1.9)	3.6 (1.5)	0.5 (−0.1 to 1.1)	0.1009	0.2 (−0.9 to 1.3)	0.7136
Hb (g l^-1^)	94.7 (12.5)	95.6 (12.8)	0.9 (−8.8 to 10.5)	0.8356	99.6 (12.4)	108.3 (11.3)	8.3 (−3.0 to 19.7)	0.1184	−12.8 (−29.8 to 4.2)	0.1146
ICP (mmHg)	28.7 (3.9)	29.4 (4.8)	−0.3 (−5.2 to 4.6)	0.8817	28.3 (3.0)	26.1 (3.0)	−2.0 (−5.7 to 1.7)	0.2160	3.3 (−2.7 to 9.3)	0.2222
*P*btO_2_ (mmHg)	5.3 (7.1)	2.4 (2.4)	−3.4 (−10.1 to 3.3)	0.2458	3.6 (5.0)	3.1 (3.9)	−1.8 (−4.3 to 0.7)	0.1113	−0.7 (−5.5 to 4.1)	0.7213

### Treatment effect on timings

**Fig 3:**
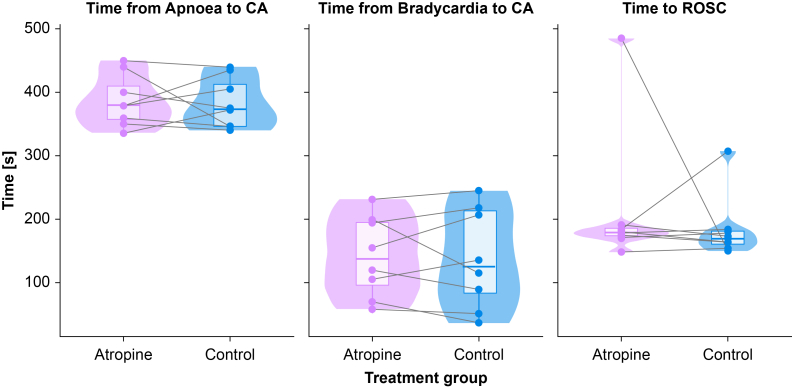
Effect of atropine on timing outcomes. Left: time from apnoea onset to CA. Middle (primary outcome): time from study-drug administration to CA. Right: time from CA to ROSC. Violin plots depict the distribution; boxes show the median (central line) and interquartile range. Points represent individual pigs, with grey lines connecting paired observations across treatments. Units are seconds; treatment groups: Atropine (purple) and Control/Placebo (blue). Y-axes are identically scaled across panels to enable direct visual comparison. CA, cardiac arrest; ROSC, return of spontaneous circulation.

For the primary outcome (time from study-drug administration to cardiac arrest), mean times were 141.5 seconds (95% CI: 83.9–199.1) with atropine *vs* 137.5 seconds (95% CI: 79.9–195.1) with placebo; the mean difference (Atropine − Placebo) was 4.0 seconds (95% CI: −32.6 to 40.6); *P*=0.803. As illustrated in Fig. 3, the direction of effect varied across animals (shorter in four pigs, longer in four).

For time from apnoea to cardiac arrest, means were 386.8 s (95% CI: 355.6–417.9) with atropine *vs* 382.4 seconds (95% CI: 351.3–413.5) with placebo; mean difference 4.4 s (95% CI: −33.8 to 42.6); *P*=0.794.

Following cardiac arrest and initiation of CPR, time to ROSC was 213.9 s (95% CI: 148.8–279.0) with atropine and 184.6 seconds (95% CI: 119.5–249.7) with placebo; mean difference 29.2 seconds (95% CI: −72.3 to 130.8); *P*=0.518.

## Discussion

In this randomised cross-over study using a porcine apnoea-induced hypoxic cardiac arrest model, atropine tended to increase heart rate but did not delay the onset of cardiac arrest. Time to arrest, systemic haemodynamics, cerebral oxygenation, and blood lactate levels were similar after atropine and placebo administration. These findings suggest no benefit from atropine in improving oxygen delivery or delaying cardiac arrest. Resuscitation outcomes, including time to ROSC, were also unaffected, confirming the limited role of atropine in hypoxia-driven arrest.^5^^,^^6^ Contrary to our hypothesis, there was no signal of harm, either.

### Context of existing data

Our data are in line with previous preclinical studies. Qian and colleagues^7^ reported that atropine accelerated cardiac arrest in hypoxic rabbits, without improving survival. Similarly, Parer and colleagues^4^ showed that atropine abolished hypoxic bradycardia in foetal sheep but did not enhance oxygen consumption. These studies support the interpretation that bradycardia in hypoxia is protective, not harmful, and blocking it may raise myocardial oxygen demand without improving tissue oxygenation. Further, Kaplan and colleagues^6^ found that bradycardia during hypoxia in rats was ‘atropine-resistant,’ while adenosine A1 receptor antagonism more effectively prolonged survival. This suggests that hypoxic bradycardia is primarily metabolic, not just vagal, and unlikely to respond to atropine alone. Consistent with our data, bradycardia due to severe hypoxia seems to be poorly responsive to anticholinergics.^5^ Of note, atropine has been withdrawn from adult asystole/PEA protocols in the 2010 edition of AHA/ACLS guidelines.^12^ In its 2025 draft scoping review, the ILCOR Paediatric Life Support Task Force withdrew prior treatment statements and concluded there is insufficient evidence to support or refute routine atropine use for bradycardia with haemodynamic compromise; instead, it issued only a good-practice statement to initiate CPR when bradycardia with poor perfusion is unresponsive to oxygenation and ventilation, and explicitly listed ‘the effect of atropine … in patients not receiving CPR’ as a key knowledge gap.^13^ Our porcine model directly addresses this gap by isolating atropine’s effect during hypoxic bradycardia preceding arrest.

Although atropine remains appropriate for vagally mediated (cholinergic) bradycardias, findings from our randomised large-animal trial suggest that its use for hypoxic bradycardia may not be appropriate. Our data did not show any physiological benefits in large mammals, and any drug administration distracts limited human resources in a crisis from the real priority, which is to restore oxygenation. Targeting bradycardia pharmacologically might be very tempting during an airway crisis, but there is no evidence that it is beneficial.

### Generalisability and limitations

Our model in young hypermetabolic, quickly desaturating pigs best mimics conditions of paediatric respiratory arrest or airway obstruction, where hypoxia precedes bradycardia and arrest. The porcine model is physiologically as close to humans as possible, but pigs are naturally less prone to developing bradycardic responses, compared to humans, due to the sympathetic hypertonus, tachycardiac response to hypoxia and fluctuating heart rate during apnoea.^14^ Of note, our protocol included pretreatment with a β-blocker to unmask hypoxic vagal responses, and this could influence the results. Both β-blockade and anaesthesia may have modified autonomic balance; while this was necessary for model stability, it could attenuate atropine’s chronotropic response.^9^^,^^10^^,^^15^ On a similar note, anaesthesia could have altered autonomic tone, and although it is unlikely, atropine might have different effects in hypoxic bradycardia when the patient is not anaesthetised, such as during most out-of-hospital severe hypoxia scenarios. We only tested a standard atropine dose in an open- label study; earlier or repeated administration might yield different outcomes, though unlikely based on existing data.^16^ Blinded preparation of identically labelled syringes with administration by an unaffiliated team member was considered but not adopted, due to staffing constraints. Observer bias on the primary outcome was further limited by the prespecified physiological definition of cardiac arrest and automated time-stamping from the digital monitoring system. Long-term outcomes were not assessed, nor were organ-level injury markers. We acknowledge that there is a significant increase in *P*btO2 between the two baselines (AM *vs* PM). We do not have another clear explanation; however, this may be related to the cross-over study design, as the probe had been inserted for a longer period during the second experimental phase. Otherwise, no carry-over effect was observed between periods, supported by the absence of a period-by-treatment interaction. Still, we believe our results mirror clinical patterns in human resuscitation. Lastly and most importantly, our study was powered to detect a 20% difference in the primary outcome (time to cardiac arrest). The fact that we did not observe any difference between the groups must not be interpreted as conclusive evidence that this difference does not exist, as our trial was designed and powered as exploratory, not an equivalence study.

### Conclusion

Atropine did not improve systemic perfusion, oxygenation, or time to ROSC in this porcine model of apnoea-induced hypoxic bradycardia. These results suggest that bradycardia in hypoxia reflects underlying oxygen deprivation rather than a reversible target for pharmacological intervention. Our findings in the context of other studies suggest that in time-critical, resource-limited airway crises where hypoxia drives bradycardia, attention and time are best spent restoring oxygenation and ventilation rather than preparing/administering atropine. With all the limitations in mind, our findings add to the body of evidence de-emphasising the role of atropine in hypoxic bradycardia during airway emergencies, during which the oxygenation, not chronotropy, determines survival.

## Authors’ contributions

Project Administration: TP

Formal analysis: PW

Investigation: TP, ZK, IO, JH, JF, RD, AV, MK

Methodology: TP, IO, AV, FD

Writing: TP, PW, MK, ZK, IO, JH, JF, RD, AV, DP, FD

Resources: AV

Conceptualisation, Supervision: FD.

All authors approved the final version of the manuscript and agree to be accountable for all aspects of the work in ensuring that questions related to the accuracy or integrity of any part of the work are appropriately investigated and resolved.

## Funding

Cooperation Intensive Care Medicine 33 Programme of Charles University and from FNKV Institutional Grant 2025. This was an investigator-initiated study, and funders are academic research institutions.

## Data Availability Statement

The datasets generated during the current study are available from the corresponding author on reasonable request.

## Declarations of interest

The authors declare no conflict of interest. The study was investigator-initiated and funded from public sources.
